# Identification of a novel mutation site in maturity-onset diabetes of the young in a Chinese family by whole-exome sequencing

**DOI:** 10.3892/mmr.2019.10464

**Published:** 2019-07-03

**Authors:** Han Yu, Jingjin Liu, Xiaofei Li, Fang Miao, Yanlan Yang

**Affiliations:** Endocrinology Department, Shanxi Provincial People's Hospital Affiliated to Shanxi Medical University, Taiyuan, Shanxi 030012, P.R. China

**Keywords:** maturity-onset diabetes of the young type 4, pancreatic and duodenal homeobox 1 gene, monogenic diabetes, biological information analysis, whole-exome sequencing

## Abstract

The aim of the present study was to determine the mutant genes and mutation sites in a family with maturity-onset diabetes of the young (MODY), in order to provide evidence for the diagnosis and treatment of clinical MODY. Based on the clinical characteristics of MODY, one family was selected from the Department of Endocrinology of Shanxi Provincial People's Hospital (Shanxi, China). The family comprised seven individuals, four of which were healthy (without MODY), and the whole exome of the individual with MODY, her father and her mother were sequenced. A suspected case (patient's uncle) and a healthy individual (patient's aunt) were sequenced for verification. The Q30 ratio was >90% in the family of three and the sequencing quality was good. The alignment rate was >95%, while the repeat sequence was <10%, with a mean sequencing depth of >120×, which is sufficient to identify mutations. According to Mutation Taster and LRT, it was predicted that the p.leu73Pro mutation of the pancreatic and duodenal homeobox 1 (PDX1) gene was deleterious. The mutation was verified by next-generation sequencing as the pathogenic site in this family. In conclusion, a novel mutation site of MODY type 4 in the PDX1 gene was identified in a family with MODY, which may provide a basis for its clinical treatment. Whole-exome sequencing appears to be of assistance in accurately diagnosing MODY.

## Introduction

Maturity-onset diabetes of the young (MODY) is an dominant, autosomal genetic disease characterized by islet function defects, insufficient insulin secretion and islet-associated antibody negativity. MODY is the most common type of monogenic hereditary diabetes (1,2). Due to its similar age at onset and clinical manifestations, MODY is often misdiagnosed as type I or atypical type II diabetes. However, different types of diabetes are characterized by different prognoses and treatment, and MODY exhibits clear family heredity. Accurate diagnosis is crucial for patients with MODY and their relatives (3).

At present, there is a lack of effective methods for the diagnosis or treatment of most hereditary diseases. With the continuous iterative update of sequencing technology and the gradual improvement in cost-effectiveness, whole-exome sequencing (WES) has become more widespread for genetically analyzing and identifying potential genetic variations that lead to disease development (4–7). In brief, WES involves the use of hybrid capture technology to obtain a DNA sequence encoding all exon regions in the genome that encode the protein, followed by high-throughput sequencing. There are as many as 180,000 exons in the human genome, accounting for ~1% of the entire genome. Exome sequencing by high-throughput sequencing technology is an effective method for identifying disease-causing genetic variations (8–11). Therefore, WES may be used to identify novel susceptibility genes for the MODY-X family of unknown genes.

In the present study, WES was performed on a family of three (two members with MODY and one healthy member), and a novel mutation site on the pancreatic and duodenal homeobox 1 (PDX1) gene was identified, which was subsequently verified by sequencing in relatives of the individuals.

## Materials and methods

### Research subjects

#### Proband

The patient was female, aged 23 years, with normal development and moderate nutritional status, who had been consuming carbonated drinks since childhood. Two years prior, the patient exhibited no hunger or an obvious incentive for eating. The patient's fasting blood glucose level was 19.0 mmol/l 2 days prior to admission to the hospital. Immediately prior to admission to the Shanxi Provincial People's Hospital on August 30, 2017, the fasting blood glucose level was 18.0 mmol/l. The patient reported no polydipsia, weight loss or blurred vision, and there was no numbness of the limbs, abdominal pain, diarrhea, fatigue or discomfort.

#### Family history

There was no history of diabetes on the maternal side of the family, whereas there were three diabetic patients among paternal relatives (grandfather, father and uncle).

#### Physical examination

On physical examination, the patient's height was 162 cm, with a weight of 56 kg and a body mass index (BMI) of 21.3 kg/m^2^; blood pressure was 122/67 mmHg. There were no obvious abnormalities of the heart, lung or abdomen, and there was no edema in either of the lower limbs.

#### Clinical data collection

The medical histories of the proband and her family members were taken into account, and their heights and weights were measured. In addition, BMI was calculated, laboratory blood glucose tests were performed, C-peptide and glycosylated hemoglobin (HbA1c) levels were measured, and liver and kidney function, blood lipids, urine microalbumin and type I diabetes-related antibodies, namely anti-islet antibody, anti-insulin autoantibody and anti-glutamate decarboxylase antibody (ICA, IAA and GAD, respectively), were assessed. Informed written consent was obtained from the patient for publication of this case report and any accompanying images, and the study was approved by the Ethics Committee of the Shanxi Provincial People's Hospital (Shanxi, China).

#### DNA extraction

A total of 2 ml venous blood was collected from three family members (two members with MODY and one healthy member). The heparin anticoagulation and SE Blood DNA kits (Omega Bio-Tek, Inc.) were used to extract peripheral blood leukocyte genomic DNA, which was then sent to Mingma Biotechnology Company for WES (12).

#### Bioinformatics analysis of exome sequence

The original sequence was quality-controlled by FastQC (version 0.11.8) (13), and BWA (version r1188) (14) software was used to align the reads to the human reference genome (hg19). The Samblaster (version 0.1.24) program (15) was used to remove duplicate reads, and GATK (version 3.8) realigner (16) was used to realign indel and Base Quality Score recalibration. To ensure the accuracy of the identification variation, five software programs (GATK, SAMtools (version 1.8) (17), FreeBayes (version 1.1.0) (18), Platypus (version 0.8.1.2) (19) and VarScan2 (version 2.4.0) (20) were used for mutation analysis and dbSNP, 1000Genomes, dbNSFP, ClinVar were used for filter mutation (4).

#### Genetic filtering

According to the patient's family history and clinical manifestations, MODY was suspected. Therefore, 14 known mutations in the MODY gene and Mendelian inheritance were analyzed, and a novel mutation site was identified on the PDX1 gene (6,21–23).

#### Polymerase chain reaction (PCR) verification

A fragment of the first exon of PDX1 was amplified by PCR and the primers were designed via the NCBI website using Primer Blast (version 3.0; http://www.ncbi.nlm.nih.gov/tools/primer-blast/). PCR was performed using the Bio-Rad T100 system (Bio-Rad Laboratories, Inc.). The PCR reaction system consisted of 1 µl DNA template [extracted using the Omega SE Blood DNA kits (Omega Bio-Tek, Inc.)], 1 µl forward (5′-CGCAGCTTTACAAGGACCCAT-3′) and reverse primers (3′-GGTGAGAACCGGAAAGGAGA-5′), 0.5 µl Taq enzyme (Takara Biotechnology Co., Ltd.), 5 µl dNTP and 4 µl 10X Ex Taq Buffer (Mg2+ free), supplemented with double-distilled water to a total volume of 50 µl. The reaction conditions were as follows: Pre-denaturation at 94°C for 3 min; denaturation at 94°C for 30 sec; annealing at 58°C for 30 sec; extension at 72°C for 45 sec, 30 cycles following 72°C extension for 5 min and, finally, the PCR product was stored at 4°C. Following the reaction, 1% agarose gel electrophoresis was performed, followed by ethidium bromide staining. The strip was observed under a UV lamp. The PCR product was sent to Shanghai Shenggong Biological Company for sequencing.

## Results

### Family diagram, clinical characteristics of family members and laboratory test results

#### Family diagram

**Figure 1.**
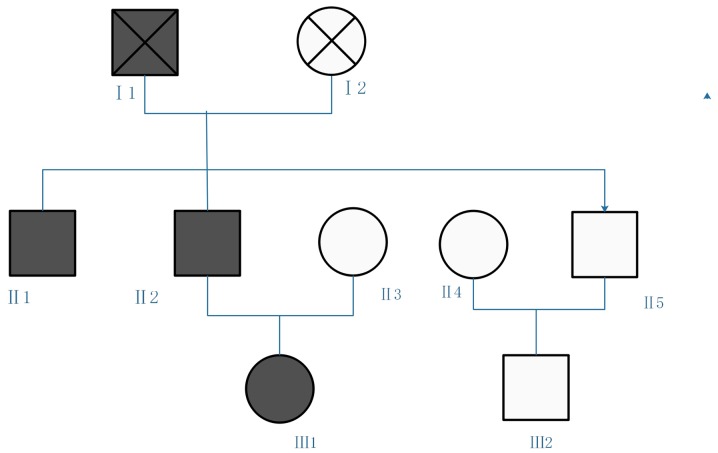
Proband family diagram. Circles represent female subject. Squares represent male subjects. White shapes represent healthy subjects. Black shapes represent patients with maturity-onset diabetes of the young.

The family included three generations. Blood samples were collected from five family members: II1, II2, II3, II4 and III1. The three diabetic patients and the other family members without clinical symptoms are presented in Fig. 1. A detailed laboratory assessment was performed on the proband (III1), and the remaining family members underwent a simple check.

#### Laboratory data and clinical characteristics

The proband's HbA1c level was 12.9%, urine sugar was 4+ and urine was negative for ketone bodies. ICA, IAA and GAD were all negative. An oral glucose tolerance test was performed simultaneously with insulin and C-peptide release experiments (Table I). There were no abnormalities in the thyroid function tests, blood lipid profile, urine microalbumin or on fundus examination.

#### Family

The proband's grandfather and mother had died, but their age and treatment for diabetes were unknown. The proband's father and uncle (II2 and II1) were diagnosed with diabetes between 35 and 40 years of age (specific age unknown), and received intermittent treatment with metformin with regular monitoring of blood glucose. In II2 and II1, the HbA1c levels were 9.5 and 9.0%, respectively. ICA, IAA and GAD were negative. The islet function test results are shown in Table I.

#### Treatment and follow-up

The proband was treated with continuous subcutaneous injection of insulin aspart using an insulin pump for 2 weeks (mean daily dose, 40 units), which was then changed to hypoglycemic treatment with subcutaneous insulin (insulin + insulin glargine) four times per day. At 3 months following discharge from the hospital, the HbA1c value was 8.8%. Injecting insulin before meals was then replaced with insulin glargine injection (14 units at bedtime) and oral agarose. At 6 months following discharge from hospital, the HbA1c level was 6.8%; insulin glargine was then replaced with oral metformin (250 mg orally three times/day) and acarbose (50 mg orally three times/day). At 9 months following discharge from hospital, the HbA1c level was 7.0%. At present, the patient's blood sugar is controlled and stable.

In terms of the father and uncle of the proband (II2 and II1), improvements in life management were important. This included control of the intake of starchy foods per meal; according to work and life conditions, starchy foods in each meal should be controlled at 100 g. Other instructions included choosing foods with a lower glycemic index, educating on the importance of exercising properly following eating for 30 min, to improve postprandial glycemic control, monitoring changes in body weight, and ensuring BMI does not exceed 25. At the 3-month follow-up, the HbA1c levels were 7.8 and 8.1%, respectively; the dose of metformin was reduced to 1,000 mg/day, and at the 6-month follow-up, the HbA1c levels were 6.8 and 7.1%, respectively. Metformin was discontinued, and blood glucose was controlled by diet and regular exercise. At the 9-month follow-up, the HbA1c levels were 6.9 and 7.0%, respectively (Table II).

#### Results of bioinformatics analysis

**Figure 2.**
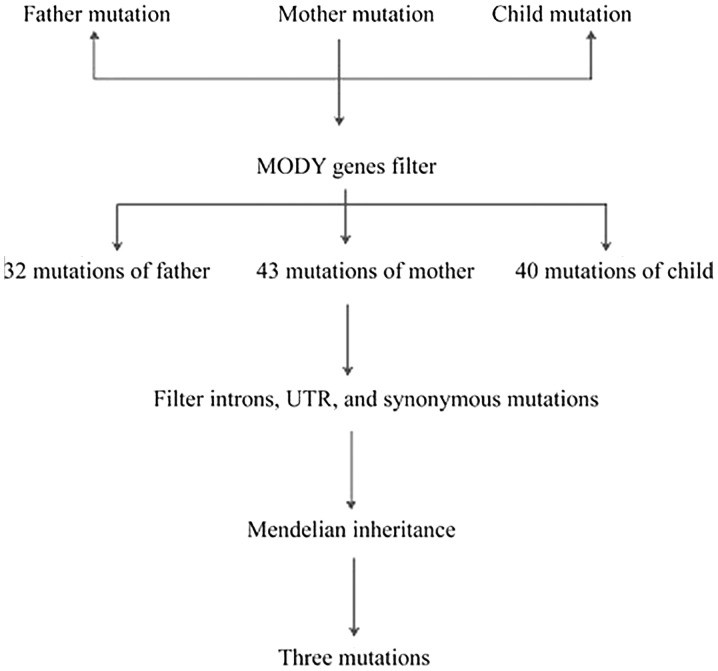
Filtering process of the pathogenic site. MODY, maturity-onset diabetes of the young; UTR, untranslated region.

The Q30 ratio based on the family was >90%. The sequencing quality was good, and the comparison rate was >95%. The repeat sequence was <10%, and the mean sequencing depth was >120×, which was sufficient to identify the mutation. The specific comparison information is shown in Table III. The father harbored 641,508 mutations, the mother had 508,998 mutations and their offspring had 490,117 mutations. The specific variation distribution is shown in Table IV. The proportion of exon regions accounted for ~20%. When filtered against 14 MODY genes, the father maintained 32 mutations, the mother maintained 43 mutations and their offspring maintained 40 mutations. Following the filtering of introns, untranslated regions and synonymous mutations, the family maintained eight mutations. According to Mendelian inheritance (filtering the site where the child genotype is consistent with the parent genotype), the remaining three sites were filtered. The entire filtration process is shown in Fig. 2 and the results are shown in Table V. The mutation rates of the ATP binding cassette subfamily C member 8 and the potassium voltage-gated channel subfamily J member 9 genes are >1% in the population. However, the frequency of mutations in the PDX1 gene has not been reported. In addition, Mutation Taster and LRT predicted that this mutation site is deleterious, the mutation site was located in the first exon, there were two exons in this gene and that this mutated amino acid was located in last transactivation domain of the protein coded by this gene. This transactivation domain contained other protein binding sites, including transcription coregulators; therefore, mutation of the transactivation domain may affect the function of the gene and may be the pathogenic factor in this family. To the best of our knowledge, that site has not been previously reported in the literature.

#### Family verification

**Figure 3.**
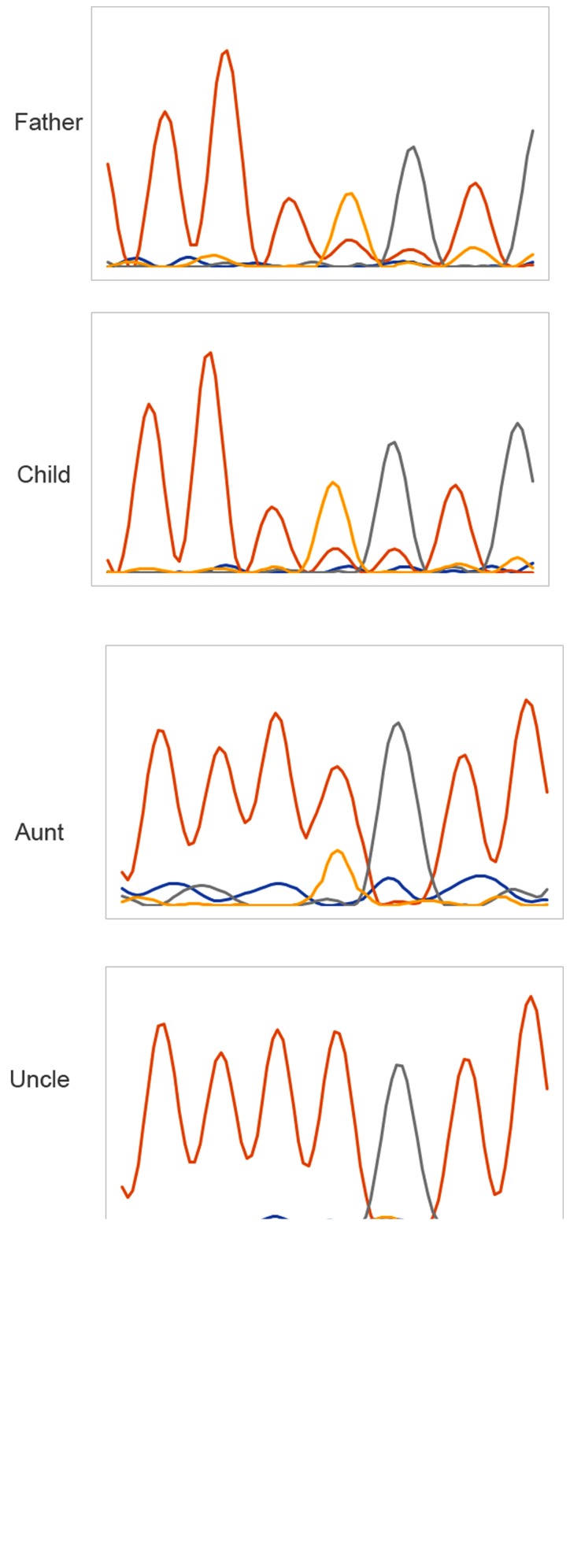
Sanger sequencing of the p.Leu73Pro site of the PDX1 gene. The father, child and uncle had the same genotype of TC, whereas the mother and aunt had the same genotype of TT. PDX1, pancreatic and duodenal homeobox 1 gene.

In order to verify whether the candidate site is a true pathogenic site, next-generation sequencing was used to analyze the information of the family and found that the uncle and the proband had the same genotype, whereas the genotype of the aunt was normal (Fig. 3).

## Discussion

MODY is a rare monogenic type of diabetes caused by heterozygous mutations of PDX1, an autosomal dominant gene that is important for regulating pancreatic function and development. The proteins encoded by PDX1 are transcriptional activators of insulin, somatostatin, glucokinase, islet amyloid polypeptide and glucose transporter 2. The encoded nuclear protein is involved in early development of the pancreas and the glucose-dependent regulation of insulin gene expression. Defects in this gene are responsible for islet hypoplasia, which may lead to early-onset insulin-dependent diabetes mellitus (IDDM) and MODY type 4 (MODY4) (24,25).

PDX1 is the main transcription factor that maintains β-cell function. During the formation of endocrine cells, the increase in PDX1 levels is crucial for the development and differentiation of pancreatic β cells. In adulthood, PDX1 is located in β and δ islet cells, and it may regulate the balance of glucose in the body by maintaining β-cell function and regulating insulin, glucose transporter 2 and glucokinase. The inhibition of PDX1 nuclear localization leads to β-cell dysfunction. Forkhead box (Fox)a2 and PDX1 are key regulators of β-cell development and function, and their mutations are associated with susceptibility to MODY, pancreatic hypoplasia and diabetes. Although Foxa2 has been shown to directly regulate the expression of PDX1 during mouse embryonic development, the effect of the regulation of this gene on postpartum β-cell maturation remains to be elucidated. Elucidating the gene regulatory network following β-cell maturation may improve current understanding of the pathological mechanisms involved in diabetes (26,27).

**Figure 4.**
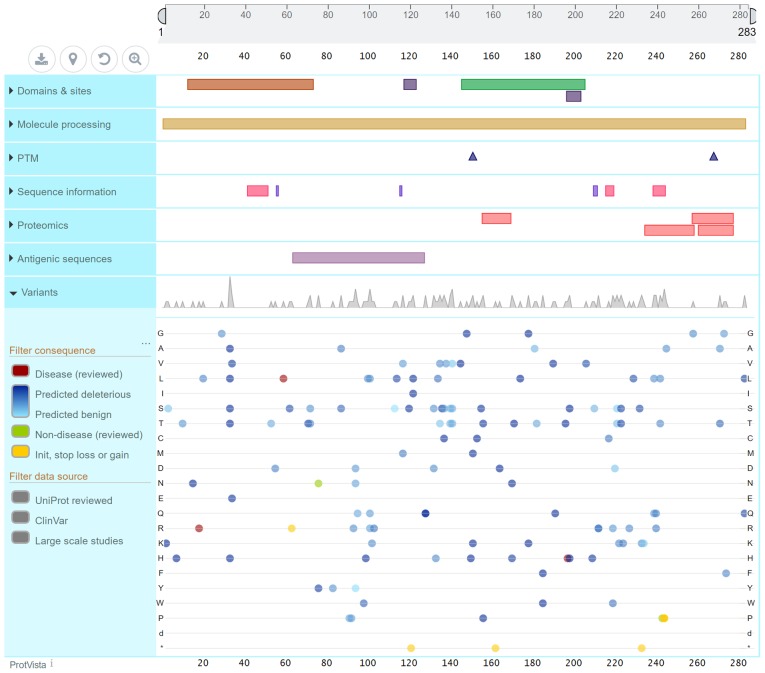
Pancreatic and duodenal homeobox 1 structure and mutation.

Previous studies have demonstrated that the genetic and acquired reduction in the expression of PDX1 may lead to type 2 diabetes and β-cell dysfunction. The PDX1 gene encodes a protein consisting of 283 amino acid residues (Fig. 4). The PDX1 protein sequence is homologous across different species. It has a transactivation domain (13–73) at the N-terminus, and the mutation site of the family in the present study was located at the end of this transactivation domain, which may affect the function of the protein. The majority of the 15 mutation sites in the ClinVar database on the PDX1 gene that resulted in MODY4 were not identified as pathogenic; however, these sites are likely pathogenic or associated with conflicting interpretations of pathogenicity. The early diabetes caused by PDX1 mutations is not associated with any signs of insulin resistance; thus, its diagnosis poses a major challenge. In this family, a novel mutation site was identified, which was considered to be a pathogenic site based on bioinformatics analysis.

MODY4 is a rare type of diabetes. The number of reports on MODY caused by gene mutations is currently limited. In the present study, the clinical manifestations of MODY4 were different. The patient age at onset was 13–67 years, with a mean age of 35 years, which was higher compared with other reported MODY ages at onset. The patients may be obese or non-obese. Insulin secretion disappeared in phase 1 and significantly decreased in phase 2 following glucose stimulation. The majority of cases have been managed with dietary modifications and oral hypoglycemic agents, with a small number of patients requiring insulin therapy.

In clinical practice, MODY is similar to type 1 diabetes in that the age of onset overlaps with adolescents, and hyperglycemia, dry mouth, polydipsia and weight loss occur. However, a difference is also notable. Patients with type 1 diabetes have high blood sugar, often accompanied by ketosis, nausea, vomiting and positivity for antibodies (ICA, IAA and GAD) in laboratory tests, the function of islets is poor and long-term insulin replacement is required. Patients with MODY generally have no ketosis. In the present study, no ketosis was present in the disease course of the proband or family members, they were negative for autoantibodies, islet function was acceptable and the hypoglycemic treatment did not require long-term insulin replacement.

In terms of MODY and type 2 diabetes, the similarities include overlap in the age of onset and negativity for antibodies, and treatment generally does not require long-term insulin; however, differences are also notable, more adolescent patients with type 2 diabetes with obesity have metabolic syndrome, and insulin function is mainly caused by insulin resistance; by contrast, patients with MODY are generally not obese and have no metabolic syndrome, and islet function defects are mainly caused by insulin secretion defects. The most marked difference between them is the genetic background, MODY exhibits multi-generation vertical inheritance, whereas type 2 diabetes exhibits multi-generation non-vertical inheritance. Therefore, patients with MODY are young, have no ketosis, have a family history of three or more generations of diabetes, and are consistent with autosomal dominant inheritance. The pathological basis is the primary defect of islet β-cell function.

In the present study, the age of the proband at onset was 23 years, and the insulin secretion curve was low, indicating poor pancreatic islet function, although there was no tendency for ketosis. In the initial stage, insulin aspart was administered via an insulin pump, and the patient's blood sugar levels quickly stabilized and were restored to normal.

Following relief of high-glucose toxicity, the insulin dose was gradually reduced. Following hospital discharge, in order to maintain stable blood sugar level, subcutaneous injection of insulin + insulin glargine was administered as hypoglycemic therapy four times per day. Following 3 months of evaluation, the HbA1c level was 8.8%, and the genetic analysis results confirmed a diagnosis of MODY4 due to a PDX1 mutation. Based on this result, the patients' subsequent hypoglycemic regimen was adjusted. Insulin before meals was discontinued, and the patient was only treated with insulin glargine injection (14 units) at bedtime, and oral acarbose at mealtimes. At 6 months following discharge from hospital, the HbA1c level was 6.8%. Insulin glargine was changed to oral metformin (250 mg orally three times/day) and acarbose (50 mg orally three times/day). The HbA1c level was 7.0% at 9 months following discharge. The comprehensive clinical manifestations and treatment outcomes were largely in line with the characteristics of MODY4.

In terms of the father and uncle of the proband (II2 and II1), their age at onset was similar (35–40 years). Following oral 75 g glucose stimulation, the secretion of insulin disappeared in phase 1. According to conventional treatment experience, such patients with poor islet function should be treated with insulin or secretagogues. However, the HbA1c levels of the father and uncle decreased to 7.8 and 8.1% following regular oral metformin administration for 3 months. Furthermore, genetic analysis revealed that both were likely to have MODY4. Therefore, consistent with previous literature on treatment experience of patients with MODY4, the dose of metformin was halved. The HbA1c levels were 6.8 and 7.1% at 6 months, and metformin was discontinued. At 9 months, the HbA1c levels were 9.9 and 7.0%. The age at onset, islet function and treatment outcomes were consistent with the characteristics of MODY4.

The findings of the present study confirmed the presence of MODY4 in families caused by PDX1 gene mutation in the Chinese population, and suggested that attention should be paid to screening for MODY among diabetic patients with a strong family genetic background and onset in adolescence. Such patients have early manifestations of insufficient insulin secretion and may be clinically misdiagnosed with type I or atypical type II diabetes. The traceability of their family history and family members' clinical characteristics is important, and precision medicine is crucial for accurate diagnosis, subsequent treatment and disease outcome. There is no uniform clinical diagnostic standard for MODY, and only genetic testing can confirm the diagnosis. With the continuous advancement of genetic testing technology, it may become easier to accurately diagnose MODY and to identify specific MODY subtypes. Furthermore, it is crucial for clinicians to design individualized treatment plans and provide genetic counseling for the relatives of such patients.

## Figures and Tables

**Table I.** Blood glucose, insulin and C-peptide levels of diabetic patients in the family.

	Blood sugar (mmol/l)			Insulin (uIU/ml)			C-peptide (ng/ml)		
			
Time (min)	III1	II2	II1	III1	II2	II1	III1	II2	II1
0	13.7	7.9	7.3	8.45	7.86	9.34	0.79	1.56	1.78
30	15.4	11.6	10.8	9.12	10.39	11.45	0.98	2.09	2.36
60	18.2	12.9	13.7	12.79	30.76	35.89	1.03	5.88	6.02
120	23.6	13.9	11.8	19.07	39.54	50.07	1.24	7.64	8.19
180	21.4	10.5	9.8	13.39	18.67	20.34	1.16	3.83	3.95

**Table II.** Follow-up results of glycosylated hemoglobin in diabetic patients in the family.

	Glycosylated hemoglobin (%)		
	
Time (months)	III1	II2	II1
0	12.9	9.5	9.0
3	8.8	7.8	8.1
6	6.8	6.8	7.1
9	7.0	6.9	7.0

**Table III.** Specific comparison information.

Quality control term	Father	Mother	Child
Total_reads	97,368,897	98,766,436	104,464,226
Q30	0.9053	0.9234	0.9353
Mapped_reads_percent	0.9689	0.9789	0.9823
Duplicated_reads_percent	0.0521	0.0773	0.0601
Mapped_average_MAQ	59.0869	59.0744	60.2534
Error_rate	0.0019	0.002	0.0021
On_target_region_reads_percent	0.7049	0.7053	0.7068
20X_coverage_bases_percent	0.9013	0.9235	0.9145
Average_coverage	122×	128×	132×

**Table IV.** Specific variation distribution.

Mutation type	Child, number of mutations	Father, number of mutations	Mother, number of mutations
DOWNSTREAM	86,953	105,783	89,108
EXON	118,407	112,863	119,841
INTERGENIC	661	42,163	775
INTRON	178,572	258,482	188,825
MOTIF	227	241	236
SPLICE_SITE_ACCEPTOR	484	321	405
SPLICE_SITE_DONOR	395	221	385
SPLICE_SITE_REGION	13,974	11,858	14,055
TRANSCRIPT	3,057	2,869	3,096
UPSTREAM	66,565	86,377	70,492
UTR_3_PRIME	12,999	13,770	13,252
UTR_5_PRIME	7,823	6,560	8,528
TOTAL	490,117	641,508	508,998

**Table V.** Candidate maturity-onset diabetes of the young mutation sites.

Chromosome	Site	Reference gene	Mutant base	Gene	Protein variation	Crowd frequency	Child genotype	Father genotype	Mother genotype
chr1	160057528	A	G	KCNJ9	p.Glu368Gly	0.04	0/1	0/1	0/0
chr11	17452500	C	T	ABCC8	p.Val560Met	0.010883	0/1	0/1	0/0
chr13	28494493	T	C	PDX1	p.Leu73Pro	–	0/1	0/0	0/0

KCNJ9, potassium voltage-gated channel subfamily J member 9; ABCC8, ATP binding cassette subfamily C member 8; PDX1, pancreatic and duodenal homeobox 1.

## Availability of materials and data

Any datasets generated and/or analyzed in the present study are available from the corresponding author on reasonable request.

## Abbreviations

MODY: maturity-onset diabetes of the young
WES: whole-exome sequencing
IDDM: insulin-dependent diabetes mellitus
MODY4: maturity-onset diabetes of the young type 4
